# Supplementary material for: Unpacking antigypsism and support for solidarity-based actions: Implications of social class, discrimination awareness, group efficacy and emotions
Source: PLoS One. 2026 Mar 11;21(3):e0340982. doi: 10.1371/journal.pone.0340982 (PMC12978468; doi:10.1371/journal.pone.0340982)
Supplement: Attachment — Submitted filename: Response to reviewes.docx [file pone.0340982.s002.docx]

PONE-D-25-25661

Unpacking antigypsism and support for solidarity-based actions: implications of social class, discrimination awareness, group efficacy and emotions

PLOS ONE

Dear Dr. Rosso,

We thank you and the reviewers for the thoughtful and constructive feedback. We have revised the manuscript accordingly. Below we provide a point-by-point response indicating the changes that we have made. We have marked these changes in the manuscript in red and with anonymized comments indicating the reviewer comments they address.

In addition, during the review process, we have made some spelling and grammatical corrections that were detected.

**Response to Reviewer #1**

We appreciate the reviewer’s positive evaluation of the intersectional design and the relevance of the topic. We address each suggestion below.

R1.#1. **The research involves exclusively non-Roma samples in Spain. The literature highlights how attitudes and dynamics of prejudice and solidarity can vary significantly across countries, regulatory contexts, and national histories. In the introduction, it would be helpful to include a reference to these possible differences on an international (or at least European) basis. The histories of Roma differ significantly from country to country, and it would be helpful to underline this aspect.**

Thank you for the suggestion. We agree. We now foreground this point at the very start in the Introduction section, where we explicitly note that these dynamics as well as their historical context vary across European countries. In the same paragraph, we have updated the discrimination data, as new information with more up-to-date data was published during the review process.

These changes can be found in lines 43-71 (pp. 3-4):

“Despite being the main ethnic minority in Europe (approximately 6 million [1]), Roma people still face *antigypsism* –negative attitudes or stereotypes attributed to Roma, whether intentional or unintentional [2]–. In 2023, 65% of European citizens surveyed acknowledged that there is widespread discrimination towards Roma in their country, 42% believed that being Roma is a disadvantage in hiring, 26% would feel uncomfortable about their child having Roma classmates at school, and 34% believed that their country's efforts to integrate Roma were ineffective [3]. However, at the European level, data on antigypsism and the historical context show substantial variation between different countries [4,5]. In Spain, the Roma population constitutes between 1% and 2.1% of the country's population, and they continue to face discrimination in different areas, such as education, employment (69% are unemployed) and housing [4]). In other countries as Hungary (7.05% of the country population), Slovakia (9.17% of the country population) or Rumania (8.32% of the country population)[6], high levels of discrimination are observed (e.g., 32% of the Roma population in Hungary and 45% in Romania are in a situation of severe material deprivation) [4].

In these countries many Roma children face discrimination at school: they are sent to “special” or Roma-only classes and schools, with fewer resources and poorer prospects, which means they have far fewer opportunities throughout their lives [4,7,8].During the Second World War, the Nazis murdered hundreds of thousands of Roma people, considering them to be “racially inferior” [9]. Later, between 1970 and 1990, approximately 90,000 Roma women were sterilized against their will in Czechoslovakia (now the Czech Republic and Slovakia) [10]. Spain presents worrying data regarding discrimination against Roma people, but it tends to present more encouraging data than the average for European countries [4]. Although its history has not been free from discrimination and persecution of the Roma population. One of the most recent events was during the dictatorship that the country experienced in the mid-20th century, when laws were used to criminalize the Roma way of life and their culture (including their language) was repressed, censored and turned into stereotypical folklore [11], with consequences that still determine the situation of the Roma today.”

R1.#2. **The methodology is very well structured and rigorous; however, it should be remembered that the manipulation of "group efficacy" in Study 2 did not produce direct effects, and an additional manipulation check would be helpful to clarify the phenomenon better. Despite the indirect analyses, the lack of a main effect suggests validity issues in the experimental manipulation. Furthermore, this undermines the reliability of the conclusions on the group efficacy variable and limits the strength of the findings on emotional mediating effects. This point would be better clarified.**

We agree that clarity on manipulation validity is crucial. We already implemented two manipulation checks in Study 2: an open-ended question about their understanding of the information presented to them, and a quantitative check evaluating perceived Group Efficacy with 1 item (To what extent is the participation of non-Roma as allies in social movements seeking equality between Roma and non-Roma effective and does it really contribute to social change?). The results showed that the manipulation significantly changed the perceived efficacy of non-Roma allies: participants in the high efficacy condition scored significantly higher than those in the low efficacy condition (*M_high_* = 5.25, *SD* = 1.56 vs. *M_low_* = 2.57, *SD* = 1.67; *t*_(998)_ = -26.20, *p* < 0.001, *d* = 1.66).

Following your comment, we have added the following paragraph to the general discussion of our manuscript (lines 642-650 on page 33):

“Regarding the absence of a direct effect of group efficacy and the participation in solidarity actions (in line with previous research as Hamann et al. [39] and Urbiola et al. [46] in some studies), our results however showed consistently that the manipulation of group efficacy worked in base of the manipulation check and operated indirectly via the targeted emotions (hope and empathy), which then predicted intentions and participation (petition signing). This path pattern is also fully consistent with previous literature on collective action, according to which beliefs about efficacy mobilize action primarily by increasing approach-oriented affect (e.g., hope) and empathic concern, rather than exerting an independent main effect [30,47,48,66–68].”

In the introduction of our manuscript, we addressed this lack of consistent results in the previous literature (lines 143-144 on page 7): “However, the main challenge for understanding the role of efficacy in social change is manipulating it, since the obtained results with this goal are inconsistent [39,46]”.

R1.#3. **Visual methodologies are interesting, but it is helpful to attach (or provide in the data repository) an exact transcription of the materials. The use of videos that manipulate accent, clothing, and speaking style risks reinforcing stereotypes rather than reducing them. A more in-depth analysis of experimental manipulations' ethical consequences and unintended side effects would be desirable.**

We thank the reviewer for this important point. We have deposited on OSF all video scripts in English (<https://osf.io/e3yzw/?view_only=b9c31687443848abbf3a5011dca2f90c>).

We have made clear in the limitations of the study that although the debriefing stated that the person in the video was an actress and the information was not true (with the exception of historical information and discrimination data), we could possibly have activated or reinforced stereotypes, and therefore there was a residual risk. This information can be found in lines 679-685 (pp. 34-35):

“Finally, audiovisual manipulations (accent, clothing, style) could activate or reinforce stereotypes, essentialize categories, and generate discomfort or be interpreted as validation of biases. To mitigate this, at the end of the questionnaire, participants were informed in detail that the person in the video was an actress and that all the contextual information she provided was fictional, except for the historical data and statistics on discrimination against Roma people, which were accurate. Information about the cover story and the real purpose of the study was also provided.”

R1.#4. **Beware of exposing the results, which suggest that discrimination can, in some cases, elicit adverse reactions or rejection (self-victimising effect against the Roma protagonist), to "victim blaming." This point, only partially discussed by the authors, should be explored more critically with respect to the risks of awareness campaigns based on storytelling or direct testimonies.**

We appreciate the important caution. We have revised the Discussion and to critically address the risks of awareness campaigns that rely on personal testimonies. Specifically, we added this paragraph on lines 612 – 626 (page 32):

“This pattern suggests a suppression effect or defensive processing [56,58], possibly reflecting that some audiences may perceive discrimination claims as illegitimate or as unduly blaming the system in the condition of discrimination awareness [59,60]. Thus, Roma target perceived discrimination can be seen as an act of “self-victimization” or complaining , triggering moral disengagement instead of promoting solidarity [59,60]. For some members of the majority group, confronting allegations of discrimination may threaten beliefs relevant to their identity (e.g., that the social system is generally fair and legitimate [64,66] and trigger defensive processing or resistance to the message [61,67]. Under these conditions, solidarity may be directly inhibited unless the same message also evokes mobilizing emotions—in particular, outrage and empathy—that help reframe the information as a moral concern and a call for solidarity-based action [30,68]. Importantly, this pattern suggest that testimonies can trigger victim-blaming; underscoring the need to design messages that channel awareness into outrage and empathy while minimizing defensiveness.”

**Response to Reviewer #2**

R2#1. **Interpretation of mixed findings for mobilizing variables: the core experimental findings for discrimination awareness and group efficacy are nuanced. While the mediation pathways (via outrage, empathy, and hope) are well-supported, the direct effects of the experimental manipulations are either non-significant (group efficacy in Study 2; discrimination awareness on participation) or negative (discrimination awareness on intentions in Study 1). The discussion would be significantly strengthened by a more direct and detailed confrontation of these null and negative direct effects. What do these inconsistent direct effects tell us about the challenges of mobilizing the non-Roma majority? I recommend refining the conclusions to emphasize that the primary mechanism of action for these variables is indirect, through the specific emotional pathways you identify. A more balanced discussion that integrates both the positive indirect and the null/negative direct effects will provide a more compelling and accurate narrative.**

We have adjusted the discussion to clearly integrate the null/negative direct effects and positive indirect effects. In summary: both awareness of discrimination and group efficacy drive solidarity action primarily indirectly, through outrage/empathy and hope/empathy, respectively; the negative direct path of awareness of discrimination on intentions in Study 1 is interpreted as a possible suppression/defensive processing effect, coexisting with a positive indirect path via outrage (we now discuss this cautiously and cite the related literature); and finally, in Study 2, the manipulation of group efficacy did strongly increase perceived efficacy (manipulation check), but its impact on action was mediated through emotions, not direct, which we also discuss more in deep now. This inconsistent or weak effect is in line with what also previous research has shown (Hamman et al., 2024; Urbiola et al, 2025). See, for example, these changes on pages 30 and 31 (lines 589 – 598):

“Furthermore, the findings reveal that the non-Roma as allies’ group efficacy is associated with solidarity-based actions primarily indirectly via hope and empathy (and correlationally, in Study 1) more than directly. The direct effects of this mobilizing variable are small or inconsistent, suggesting that their main role is to create psychological conditions under which mobilizing emotions can emerge (outrage in Study 1; outrage and empathy in Study 2), rather than to directly increase solidarity-based action by themselves. Future research with a more extend and complex manipulation of group efficacy in line with Hamann et al. [39] suggestions (including social norms, agency for achieving goals more directly, etc.) could contribute to demonstrate if and with which conceptualization group efficacy can directly mobilize allies.”

On page 31 (lines 612-614):

“Across studies, the direct patterns of the mobilizing variables on solidarity-based action –intentions and participation– are mixed, but highly consistent in their indirect path via emotions.”

And on page 34 (lines 670-677):

“At the same time, the weak, null or negative (for discrimination awareness) direct paths for group efficacy and discrimination awareness underscore a crucial challenge for mobilizing the non-Roma majority, where informing people about discrimination or emphasizing that they can be effective allies could not be enough, at least in the ways that we are testing it and in this intergroup context. Mobilization can require messages that activate the concrete emotional responses while minimizing defensiveness and threat to system justification beliefs.”

R2#2. **Theoretical implications of the suppression effect: the potential suppression effect in Study 1 is a fascinating finding. The brief mention of "self-victimization" is a plausible starting point, but this section should be expanded. Please consider integrating theories of backlash, system justification, or perceived illegitimacy of claims to provide a deeper theoretical explanation for why simply being made aware of discrimination might, for some, directly inhibit solidarity.**

We have added further explanations of this effect in the Discussion section to avoid reducing this to self-victimization. It can be found on lines 620-634 (page 32):

“This pattern suggests a suppression effect or defensive processing [56,58], possibly reflecting that some audiences may perceive discrimination claims as illegitimate or as unduly blaming the system in the condition of discrimination awareness [59,60]. Thus, Roma target perceived discrimination can be seen as an act of “self-victimization” or complaining , triggering moral disengagement instead of promoting solidarity [59,60]. For some members of the majority group, confronting allegations of discrimination may threaten beliefs relevant to their identity (e.g., that the social system is generally fair and legitimate [64,66] and trigger defensive processing or resistance to the message [61,67]. Under these conditions, solidarity may be directly inhibited unless the same message also evokes mobilizing emotions—in particular, outrage and empathy—that help reframe the information as a moral concern and a call for solidarity-based action [30,68]. Importantly, this pattern suggest that testimonies can trigger victim-blaming; underscoring the need to design messages that channel awareness into outrage and empathy while minimizing defensiveness.”

R2#3. **Clarity on statistical model specification: for full rigor and reproducibility, please clarify the exact specification of the mediation models. The captions for Figures 2 and 4 state that analyses were performed "including Social Class Condition" but that it was omitted from the figures. To ensure readers can fully interpret your results, please state explicitly in the method or results section whether the coefficients for discrimination awareness and group efficacy in the mediation models (e.g., in Tables 2 and 4) were derived from models that controlled for the other experimental factors (social class, and in Study 2, the other main factors) as covariates in all paths of the model.**

Thank you for this helpful suggestion. We have added a new “Analytic strategy” subsection in the Method section (pp. 13-14, lines 302-313 and p. 23, lines 486-488) that specifies the exact mediation models and variables included for each analysis. For readability, the figures omit Social Class paths, but the text and table notes make this specification explicit. We also clarify our reporting conventions (unstandardized B, SE, 95% CI, *p*) and that indirect effects were tested using 10,000 bootstrap resamples with 95% CIs. No other additional variables were included as covariates (e.g., age, gender, political orientation) in these mediation models.

In the previous version, the following sentences were added regarding the mediation analysis performed:

For Study 1 (lines 351-357 on pp. 16-17):

“However, when mediation analyses with emotions were performed with 10,000 bootstrapping samples using GLM Mediation Model on Jamovi, indirect effects were found. We carried out a model with the two main independent variables [discrimination awareness (X_1_) and social class (X_2_)] as predictors, the emotions towards the situation of Roma [outrage (M_1_) and hope (M_2_)] and towards Roma people [pity (M_3_) and empathy (M_4_)] as mediators, and solidarity-based actions on intentions (Y_1_) and participation (dichotomous) (Y_2_) as dependent variables (see Fig 2 and Table 2).”

For Study 2 (lines 535-541 on page 27):

“Mediation analyses were performed using GLM Mediation Model on Jamovi, using 10,000 bootstrap samples. We examine whether outrage (M_1_) and hope (M_2_) towards the situation of Roma, and empathy (M_2_) and pity (M_3_) emotions towards Roma people will mediate the relationship between discrimination awareness (X_1_), group efficacy (X_2_), or social class (X_3_) –entered simultaneously as predictors in all paths of the model– and solidarity-based actions intentions (Y_1_) and participation (dichotomous) (Y_2_) (see Fig 4 and Table 4).”

As mentioned, following your recommendation, we have added the following paragraphs. On pages 13-14 (lines 302-313) for Study 1:

**“Analytic strategy**

To examine effects on prejudice towards Roma, we estimated a between-subjects multivariate general linear model (MANOVA) with the two experimental factors entered as independent variables (Social Class: low vs. high; Discrimination Awareness: low vs. high).

We tested mediation models entering simultaneously as independent variables (X): Social Class Condition (low = 0; high = 1) and Discrimination Awareness Condition (low = 0; high = 1). The mediators (M) were the emotion variables (outrage, empathy, hope and pity), and the outcomes (Y) were solidarity-based action intentions and participation (petition signing). We report unstandardized estimates (B), Standard Error (SE) and 95% CIs. Indirect effects are based on 10,000 bootstrapping; significance is inferred when the 95% CI excludes zero.”

On page 23 (lines 486-488) for Study 2:

**“Analytic strategy**

The analyses were performed in the same way as in Study 1. In this study, Group Efficacy (low = 0; high = 1) was also added as a predictor variable (X).”

In addition to adding these sections, the caption under the mediation figures has been modified [e.g., Model includes Social Class and Discrimination Awareness simultaneously as predictors on all paths (a, b, c′). However, the paths for Social Class Condition are not shown to clarify the figure due to not significant effects and explicitly said in the notes of the figure].

Finally, we have been contacted by the journal to add clearer information about informed consent. We have revised the ‘Methods’ section and now specify that all participants gave their informed consent, that this consent was obtained in writing in electronic format (participants had to give their express consent before accessing the questionnaire), and that all participants were adults (over 18 years of age). Since no minors participated in the study, parental or guardian consent was not required. Find this new information on page 11 (lines 240-243) and 22 (lines 467-471).

We would like to thank you once again for your constructive and insightful comments, which have greatly helped us to improve the quality and clarity of the manuscript.

We hope that the revised version meets your expectations and we remain at your disposal for any further clarifications.

Sincerely,

The authors
